# Termite Nest Associated *Bacillus siamensis* YC-9 Mediated Biocontrol of *Fusarium oxysporum* f. sp. *cucumerinum*

**DOI:** 10.3389/fmicb.2022.893393

**Published:** 2022-06-01

**Authors:** Lingfeng Zhou, Junyong Wang, Fei Wu, Caiping Yin, Ki Hyun Kim, Yinglao Zhang

**Affiliations:** ^1^College of Life Sciences, Anhui Agricultural University, Hefei, China; ^2^School of Pharmacy, Sungkyunkwan University, Suwon, South Korea

**Keywords:** *Bacillus siamensis*, *Fusarium oxysporum* f. sp. *cucumerinum*, antifungal activity, C_15_-surfactin, biocontrol

## Abstract

The antagonistic potential of bacteria obtained from the nest of *Odontotermes formosanus* was assessed against *Fusarium oxysporum* f. sp. *cucumerinum* (FOC). Of 30, seven termite nest-associated bacteria strains had biocontrol potential. Among them, the strain YC-9 showed the strongest antifungal activity toward FOC. Phylogenetic analysis of the 16S rRNA amplified product of YC-9 revealed its identification as *Bacillus siamensis*. The *in vivo* antifungal activity experiment showed that the application of YC-9 at 10^8^ cfu/ml significantly reduced the cucumber wilt incidence with a control efficacy of 73.2%. Furthermore, plant growth parameters such as fresh weight, dry weight, plant height, and root height were significantly improved by 42.6, 53.0, 20.8, and 19.3%, respectively. We found that inoculation with *B. siamensis* YC-9 significantly increased the activity of defensive enzymes such as peroxidase (POD), polyphenol oxidase (PPO), and phenylalanine ammonia-lyase (PAL) in diseased cucumber roots, thereby raising the resistance. PCR using gene-specific primers revealed that *B. siamensis* YC-9 contains biosynthetic genes for known antibiotics, including bacillomycin, iturin, and surfactin. Chemical analysis of the cultivation of *B. siamensis* YC-9 resulted in the isolation of five metabolites, including hexadecanoic acid (**1**), cyclo-(L-phenylalanylglycine) (**2**), cyclo-(L-trans-Hyp-L-Leu) (**3**), C_15_-surfactin (**4**), and macrolactin A (**5**), the structures of which were identified by the analysis of NMR spectroscopic data and MS. Among them, the compound **4** showed significant antifungal activity against conidial germination of FOC with an IC_50_ value of 5.1 μg/ml, which was comparable to that of the positive control, cycloheximide (IC_50_ value of 2.6 μg/ml). Based on these findings, this study suggests that termite-nest associated *B. siamensis* YC-9 could be a potential biological control agent for integrated control of soil-borne diseases like cucumber *Fusarium* wilt.

## Introduction

Cucumber (*Cucumis sativus* L.) is one of the most important economic crops worldwide, and it is popular and favored by consumers for its distinct aromas and flavors (Shan et al., 2021). It is well known for its softness and succulence and contains a variety of nutrients, such as potassium, copper, manganese, phosphorus, pantothenic acid, dietary fibers, and vitamins (Cao et al., 2020). However, cucumber is susceptible to many pathogens (Lebeda et al., 2007). Cucumber *Fusarium* wilt, induced by the pathogen *Fusarium oxysporum* f. sp. *cucumerinum* (FOC), is a typical soil-borne fungal disease and also one of the most important cucumber diseases worldwide (Cao et al., 2020). The disease could reduce 10–30% of cucumber production and cause quality degradation, which results in serious economic losses (Li et al., 2009; Shi et al., 2016).

Chemical controls can effectively protect plants from infectious pathogens (Zhang et al., 2018). However, the drawbacks of chemical fungicides are obvious when pathogen resistance to pesticides, food safety, and environmental quality are considered. As an efficient, environmentally friendly, and sustainable method, biocontrol is used to protect plants against soil-borne diseases (Ben Abdallah et al., 2016; Koch et al., 2018; Yadav et al., 2021).

Many microbial strains are currently used as biological control agents (BCAs) (Chen et al., 2020), including yeast, bacteria, fungi, and actinomycetes (Sharma et al., 2009; Wisniewski et al., 2016; Sujarit et al., 2020). In the past, BCAs were mainly isolated from habitats such as soil, and it is currently difficult to isolate new strain resources due to long-term repetitive studies (Saxena et al., 2020). Therefore, to discover new BCA resources, it is necessary to find new microbial resources in special habitats. As a biological material, termite nest is an important microbial resource. The *Streptomyces* of termites’ feces in an underground nest could produce a series of bioactive metabolites that provided termites with a certain degree of protection against some insect pathogenic fungi (Chouvenc et al., 2018). For example, Oberpaul et al. (2020) isolated, screened, and identified new species with the potential of anti-pathogenic fungi from termite nests by high-throughput culture. However, there are few reports on termite nest-related microorganisms.

In this study, we evaluated the ability of *Bacillus siamensis* YC-9, identified from among 30 isolates obtained from the nest of *O. formosanus*, to function as a plant growth promoting bacterium and control cucumber *Fusarium* wilt. To investigate the mechanism of its activity, an induced resistance experiment was designed to detect changes in the enzyme activities of resistance-related enzymes in cucumber roots. We also performed an examination of known antibiotic related genes, and further attempts were made to isolate and identify the active metabolites.

## Materials and Methods

### Bacteria Isolation From Termite Nest

Nests of *O. formosanus* were collected in July 2017 in Jiangyin City, Jiangsu Province, China. The termite was authenticated by Professor Jianguo Wang (Department of Plant Protection, College of Agriculture, Jiangxi Agricultural University). More than three termite nests were grinded in mortar, followed by the treatment with an equal volume of 70% (v/v) ethanol for 4 h at room temperature to kill vegetative cells. Subsequently, the material was washed three times with sterile water. The diluted sample was coated onto the Luria-Bertani (LB) culture medium (NaCl 10 g, peptone 10 g, yeast extract 5 g, and agar 15 g, in 1 L of distilled water), which was supplemented with 0.1% sodium taurocholate in order to promote spore germination (Browne et al., 2016). All mediums were cultured at 37°C for 24 h. After 2 days, a total of 30 germinal microorganisms were conserved on slant LB medium and stored at 4°C.

### Screening of Bacterial Isolates for Antifungal Activity Against *Fusarium oxysporum* f. sp. *cucumerinum* and Antifungal Spectrum Efficacy of YC-9

All the bacteria isolates were initially screened to assess their antifungal activity against *F. oxysporum* f. sp. *cucumerinum* (FOC) using a dual culture method on potato dextrose agar (PDA) plates (Ribeiro et al., 2021). A single 6 mm plug of the pathogen from 4 days of growth was inoculated in the center of the PDA medium. The isolates with 1 μl bacterial suspension (10^8^ cfu/ml) were spot inoculated at three sites equidistant from the center of Petri plates. PDA media inoculated with the pathogen alone were used as control. All the plates were cultured continuously at 28°C. After 4 days, the diameter of the pathogen colony (cm) was recorded. The inhibition percentage (I) was calculated using the following formula (Nasr et al., 2019):

I (%) = [(R – r)/R] × 100

where r and R are the radial growth of fungal pathogens in dual plate culture and control plates, respectively.

The antifungal spectrum of the selected strain YC-9 against the following plant pathogens was detected by the dual culture method described above: *F. oxysporum* f. sp. *vasinfectum*, *Alternaria solani*, *Colletotrichum graminicola*, *Curvularia lunata*, *Corynespora cassiicola*, *F. oxysporum* f. sp. *mornordicae*, *Botrytis cinerea*, and *Fusarium graminearum*. All plant fungal pathogens were obtained from the Department of Microbiology, School of Life Sciences, Anhui Agricultural University, Hefei, China.

### Identification of Antagonistic Isolates

The antagonistic isolates were identified using 16S rRNA gene sequences and phylogenetic analysis (Gorai et al., 2021). Total DNA was extracted using an Aidlab DNA extraction kit (Aidlab Biotechnologies Co., Ltd., Beijing, China). Primers 27F (5′-AGAGTTTGATCATGGCTCAG-3′) and 1492R (5′-GGTTACCTTGTTACGACTT-3′) were used as amplification primers. Polymerase chain reaction amplification (PCR) was utilized on the basis of the following steps: pre-denaturation at 94°C for 5 min, denaturation at 94°C for 30 s, annealing at 55°C for 30 s, extension at 72°C for 90 s, and a final extension at 72°C for 10 min, with 30 cycles in total. DNA sample was sequenced by General Biol (Anhui) Co., Ltd. Additionally, 16S rRNA gene sequence-based phylogenetic analysis was performed by the Maximum Likelihood method with the MEGA 6.0 software. A bootstrap analysis with 1,000 replicates was performed to calculate node support.

### *In vivo* Antifungal Activity in Pot Experiments and Plant Growth Characteristics

#### *In vivo* Antifungal Activity in a Pot Experiment

Plump and uniform cucumber seeds (Jinyan No. 4) were selected and soaked with 2% NaClO for 10 min, then soaked with 75% alcohol for 30 s, and washed three times with sterilized water. The seeds were then sowed in plastic cups (50 × 50 × 70 mm) filled with sterilized soil. After 15 days, 20 ml of YC-9 suspension (10^8^ cfu/ml) was slowly injected into the cucumber rhizosphere. The same volume of sterile water was added as a control. There were 25 repeats of the treatment and control groups. After 5 days, 20 ml of FOC conidial suspension (10^5^ spores/ml) was inoculated in the rhizosphere of all cucumber seedlings. The planted cups were placed in an illumination incubator at 28°C with light (12 h)/dark (12 h) cycles. At 15 days after fungal inoculation, wilt development in each cucumber plant was rated using the Yang et al. (2011) scale: 0 (whole plant was healthy); 1 (<10% of leaves wilted); 2 (11–20% of leaves wilted); 3 (21–50% of leaves wilted); 4 (50–100% of leaves wilted); and 5 (the plant was dead). The disease index was transformed to percent disease index (PDI) before analysis of variance. The disease indices and biocontrol efficiency were calculated according to the following formulas:

Percent disease index (%) = Σ (rating × number of plants rated)/(total number of plants × highest rating) × 100

Biocontrol efficiency (%) = (disease index of control – disease index of treated)/disease index of control × 100

#### Plant Growth Parameters Study

The preliminary preparation of the experiment is the same as that in the “*In vivo* Antifungal Activity in a Pot Experiment” section. After 30 days of inoculation with YC-9 or sterile water, the plant growth characteristics were measured by the modified method developed by Wang et al. (2020). The heights of the aerial and underground parts were measured as the plant and root height, respectively. After washing the root soil and removing the surface moisture with absorbent paper, the cucumber seedlings were weighed to get the fresh weight. The dry weight was weighed after the seedlings were dried at 65°C for 24 h.

### Assessment of Systemic Resistant Enzymes in the Cucumber Roots

After 15 days of cultivation, the cucumber seedlings were inoculated with different treatments, including sterile water (A), YC-9 suspension (B), FOC conidia suspension (C), and challenge inoculation with FOC after 48 h of YC-9 suspension inoculation (D). The experiment was set up for three replicates. An amount of 0.2 g of root samples was collected on the 2nd, 4th, 6th, 8th, and 10th days after inoculation and homogenized with liquid nitrogen. The homogenized tissues were rinsed with 1 ml of ddH_2_O (25 mM borate buffer for the PAL assay) at 4°C. The tissue extracts were centrifuged at 5,000 × *g* for 5 min at 4°C. The supernatant was stored at –80°C for the enzymatic activity assays.

#### Determination of Phenylalanine Ammonia-Lyase Activity

The PAL activity was measured according to the modified method of Mori et al. (2001). A volume of 200 μl of enzyme extract was mixed with 2 ml of 0.15 mM borate buffer (pH 8.8) and 0.8 ml of 56.25 μM L-phenylalanine and incubated at 37°C for 30 min. A volume of 0.1 ml of HCl at a concentration of 5 M was added to terminate the reaction. The enzyme activity was measured spectrophotometrically at 290 nm. The unit of enzyme activity was determined as the amount of plant fresh weight that was required to change the optical density by 1.0 for 1 h.

#### Determination of Polyphenol Oxidase Activity

The activity of PPO (EC 1.10.3.2) was determined by the modified method developed by Cavalcanti et al. (2007). A volume of 50 μl of enzyme extract was mixed with 1.5 ml of 0.2 M pyrocatechol and 1.5 ml of 0.05 M phosphate buffer (pH 6.8) and further incubated at 30°C for 2 min. The enzyme activity was measured spectrophotometrically at 398 nm. The unit of enzyme activity was determined as the amount of plant fresh weight required to change the optical density by 1.0 for 1 h.

#### Determination of Peroxidase Activity

The activity of POD was determined by the method described by Choudhary (2011) with little modification. A volume of 20 μl of enzyme extract was mixed with 1.95 ml of 0.2 M acetate buffer (pH 5.0), 1 ml of 0.1% O-methoxyphenol, and 1 ml of 0.08% hydrogen peroxide, and incubated at 25°C for 1 h. The peroxidase (POD) activity was measured spectrophotometrically at 470 nm. The unit of enzyme activity was determined as the amount of plant fresh weight required to change the optical density by 1.0 for 1 h.

### Detection of Encoding Genes of Known Antibiotics

The detection of encoding genes of known antibiotics was determined by the method described by Gorai et al. (2021) with less modification. A PCR reaction was carried out in the mixture, which contained 1 μl (50 ng) of DNA template of YC-9, 1 μl of each primer (which was listed in Table 1), 12.5 μl of Premix Taq (LA Taq Version 2.0), and 9.5 μl of ddH_2_O. PCR amplification was utilized on the basis of the following steps: pre-denaturation at 94°C for 10 min, denaturation at 94°C for 45 s, annealing at 55°C for 45 s, extension at 72°C for 2 min, and a final extension at 72°C for 10 min, with 35 cycles in total. The amplified product was subjected to electrophoresis with 1% agarose gel. The results were observed and recorded by a gel imager.

**TABLE 1:** Primers used for *B. siamensis* YC-9 antibiotic encoding genes amplification.

	Genes	Primers	Sequences (5′–3′)	PCR product size expected/detected (pb)	References
Surfactin	*srf*AA	SRFAF	AAAGGATCCAGCCGAAGGGTGTCATGGT	1,300/yes	Alvarez et al., 2012
		SRFAR	AAAAAGCTTGTTTTTCTCAAAGAACCAGCG		
Fengycin	*fen*D	FEND1F	TTTGGCAGCAGGAGAAGTTT	964/no	Stankovic et al., 2012
		FEND1R	GCTGTCCGTTCTGCTTTTTC		
Bacillomycin	*bmy*B	BACC1F	GAAGGACACGGCAGAGAGTC	875/yes	Stankovic et al., 2012
		BACC1R	CGCTGATGACTGTTCATGCT		
Iturin	*itu*C	ItuC-F	AGGATCCAAGCGTGCCTTTTACGGGAAA	465/yes	Alvarez et al., 2012
		ItuC-R	AAAAAGCTTAATGACGCCAGCTTTCTCTT		
DAPG	*phl*D	Phl2a	GAGGACGTCGAAGACCACCA	745/no	Velusamy et al., 2006
		Phl2b	ACCGCAGCATCGTGTATGAG		
PRN	*prn*C	PrnCf	CCACAAGCCCGGCCAGGAGC	719/no	Bakthavatchalu et al., 2013
		PrnCr	GAGAAGAGCGGGTCGATGAAGCC		

### Isolation and Identification of Secondary Metabolites From YC-9

The fermentation of strain YC-9 was conducted according to the methods described in detail previously (Liu and Sun, 2021). The single colony of *B. siamensis* YC-9 strain was inoculated into a 250-ml flask containing 100 ml of LB medium and cultured at 180 r/min for 24 h at 28°C. Then, 5 ml of this seed culture was inoculated into a 1-L flask containing 500 ml of LB medium (× 39) and cultured at 180 r/min for 3 days at 28°C. The culture broth (19.5 L) was filtered by gauze to afford the supernatant. The supernatant was extracted with EtOAc (3 × 20 L) at room temperature. The EtOAc phase was evaporated *in vacuo* to afford a crude extract and then subjected to silica gel column elution with a stepwise gradient of CH_2_Cl_2_/MeOH (100:0–100:8, v/v) to give five fractions (Fr1-Fr5). Fr1 (CH_2_Cl_2_/MeOH, 100:0, v/v) was further fractionated on a silica gel column, eluting with a stepwise gradient of petroleum ether (PE)/ethyl acetate (EA) (100:0–100:10, v/v) to give compound **1** (9.5 mg). Fr3 (CH_2_Cl_2_/MeOH, 100:2, v/v) was repeatedly chromatographed over a silica gel column to give compound **2** (22.7 mg). Fr5 (CH_2_Cl_2_/MeOH, 100:8, v/v) was repeatedly chromatographed over a silica gel column to give compound **3** (46.8 mg). The remaining part of Fr5 was loaded onto a Sephadex LH-20 column (100% MeOH) to yield compounds **4** (38.4 mg) and **5** (6.1 mg). Due to the limitation of separation means, no monomer compound was isolated and purified from Fr2 and Fr4.

Structural identifications of the metabolites were made on the basis of the spectroscopic data and mass spectrometry. Nuclear magnetic resonance (NMR) spectra were recorded on an Agilent II DD2 instrument operating at 600 MHz for ^1^H and 150 MHz for ^13^C, while the DEPT and 2D spectra (COSY, HMQC, and HMBC) were obtained using the standard Agilent software. Chemical shifts were given in parts per million (δ) downfield from the TMS internal standard. The electrospray ionization mass spectrometry (ESI–MS) spectra were determined on a micrOTOF II mass spectrometer (Mariner Mass 5304, United States).

### Assessment of the Antifungal Activity of Secondary Metabolites

The inhibitory activities of the secondary metabolites of strain YC-9 against spore germination of FOC were assessed as reported previously with slight changes (Zhang et al., 2013). FOC spores were obtained from PDA plates of 5-day-old cultures. All secondary metabolites were made to obtain solutions with serial concentrations in aqueous solution (1% acetone). A tested compound solution (60 μl) was added to a spore suspension (60 μl, 10^5^ spores/ml). Then, aliquots of 40 μl of spore suspension from each were placed on separate glasses in triplicate. Cycloheximide (Hefei Bomei Biotechnology Co., Ltd.) was used as the positive control. The negative control was treated with the above aqueous solution alone. Slides containing spores were incubated in a moisture chamber at 28°C for 6 h, after which approximately 100 spores were examined under a light microscope to determine the percentage of germinated spores. The percentage of spore germination inhibition was calculated from mean values as follows:

Inhibition (%) = (A – B)/A × 100

where A and B are the percentages of germinated spores in the control and the sample, respectively.

### Statistical Analysis

The contribution and significance of the treatments were determined using a one-way analysis of variance (ANOVA) followed by Duncan’s multiple range test or Student’s *t*-test to determine significant differences between treatment means (*p* < 0.05) with the SPSS 20.0 software. All experiments were performed with at least three independent replicates, unless otherwise stated.

## Results

### Screening of Antagonistic Bacteria Against *Fusarium oxysporum* f. sp. *cucumerinum* and Antifungal Spectrum of YC-9

**FIGURE 1:**
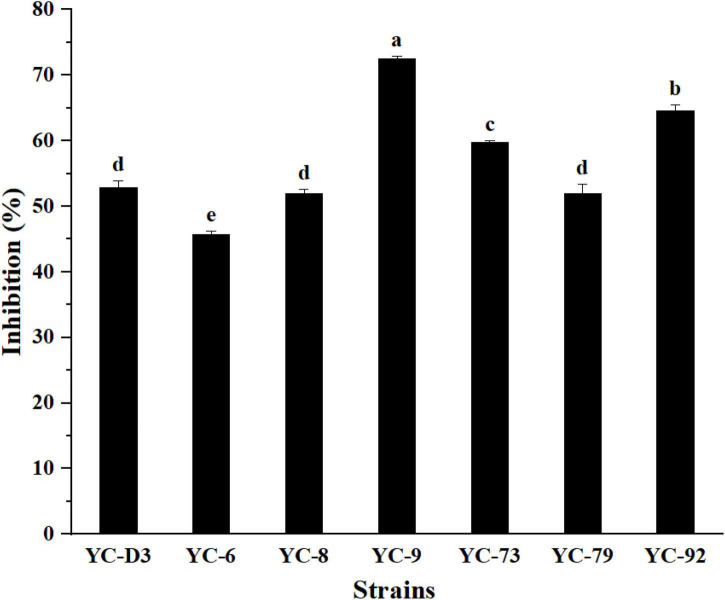
The inhibitory efficiency of seven bacterial strains against FOC. Bars with the same letters are not significantly different according to Duncan’s multiple range test at *P* < 0.05. Error bars represent the standard deviation from three replicates.

In total, 30 bacterial strains were isolated from the nest of the termite, *O. formosanus*, seven of which exhibited strong antifungal activity against FOC with the inhibition rates ranging from 45.7 to 72.5% in the dual culture (Figure 1). Among them, the strain YC-9 showed the strongest antifungal activity against FOC, with an inhibition rate of 72.5% (Supplementary Figure 1). Therefore, the strain YC-9 was selected as the objective strain in this study to further investigate its biocontrol effect.

**FIGURE 2:**
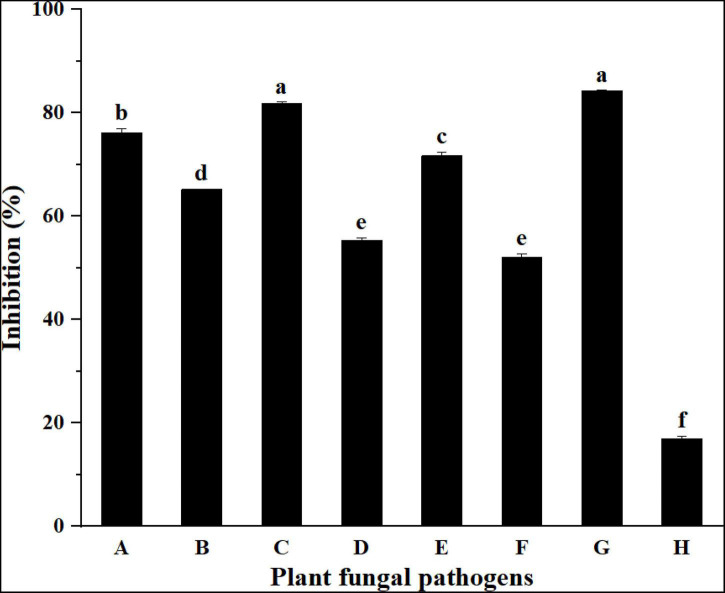
Inhibition activities of strain YC-9 against plant fungal pathogens. A: *F. oxysporum* f. sp. *vasinfectum*; B: *A. solani*; C: *C. graminicola*; D: *C. lunata*; E: *C. cassiicola*; F: *F. oxysporum* f. sp. *mornordicae*; G: *B. cinerea*; H: *F. graminearum*. Bars with the same letters are not significantly different according to Duncan’s multiple range test at *P* < 0.05. Error bars represent the standard deviation from three replicates.

The inhibitory activities of the strain YC-9 against eight selected pathogenic fungi are presented in Figure 2 and Supplementary Figure 2. The results showed that the strain YC-9 had the most potent inhibitory effect on all tested fungi except for *F. graminearum*. The strain YC-9 was found to have broad spectrum antifungal activity and could be applied to biological control of other crop diseases.

### Identification of the Strain YC-9

**FIGURE 3:**
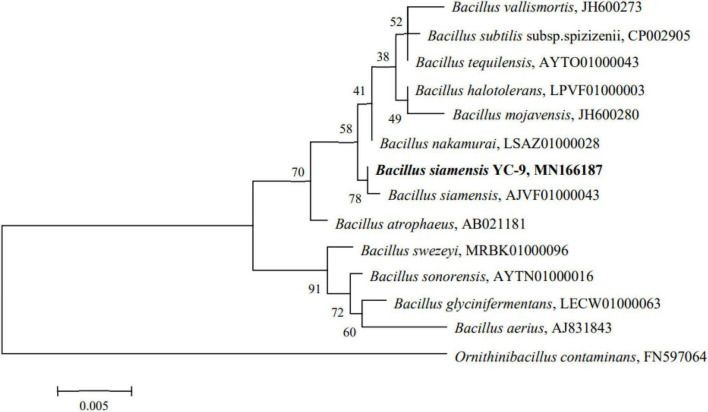
Phylogenetic tree of strain YC-9 based on maximum-likelihood method analysis of 16S rRNA sequences data.

A 1,431 base pair (bp) strain of the 16S rRNA gene was amplified and sequenced. The sequence was then queried against the EzBioCloud database. Relevant type strains were selected to construct a phylogenetic tree using the maximum-likelihood method (Kumar et al., 2021). The phylogenetic analysis revealed that the strain YC-9 and *B. siamensis* clustered on the same branch, with 99.86% sequence similarity for the 16S rRNA gene sequence (Figure 3), indicating the strain YC-9 as *B. siamensis*.

### Biocontrol Efficacy of *Bacillus siamensis* YC-9 Against Cucumber *Fusarium* Wilt

*In vivo* challenge experiment was performed to check the ability of *B. siamensis* YC-9 to reduce the disease severity. After 20 days of treatment with conidial suspension of FOC (10^8^ cfu/ml), 32.8% disease severity was observed for control. However, only 8.8% of disease severity was recorded due to the application of *B. siamensis* YC-9 even after pathogenic treatment (Supplementary Figures 3, s4). The calculated control efficacy was 73.2%. This observation strongly suggested that the *B. siamensis* YC-9 had significant suppression activity against FOC even after treatment with the pathogenic conidia in high concentration.

### Effects of *Bacillus siamensis* YC-9 on Plant Growth Characteristics

The effects of inoculation with *B. siamensis* YC-9 on cucumber growth characteristics are presented in Table 2. The results showed that *B. siamensis* YC-9 increased the fresh weight by 42.6%, dry weight by 53.0%, plant height by 20.8%, and root length by 19.3%. Thus, the application of *B. siamensis* YC-9 could promote cucumber growth in comparison with those grown without the experimental strain.

**TABLE 2:** Effect of *B. siamensis* YC-9 on growth of cucumber seedlings.

Treatment	Fresh weight (g)	Dry weight (g)	Plant height (cm)	Root length (cm)
*B. siamensis* YC-9	16.34 ± 4.26*	1.76 ± 0.22*	32.68 ± 5.77*	15.60 ± 3.43*
CK	11.46 ± 3.55	1.15 ± 0.24	27.05 ± 3.17	13.08 ± 2.29

Data represent means ± standard deviation. Asterisks indicate a significant difference between the control (CK) and B. siamensis YC-9 treated plants (t-test; *p < 0.05).

### Induction of Resistance-Related Enzymes in the Roots of Cucumber Seedlings

**FIGURE 4:**
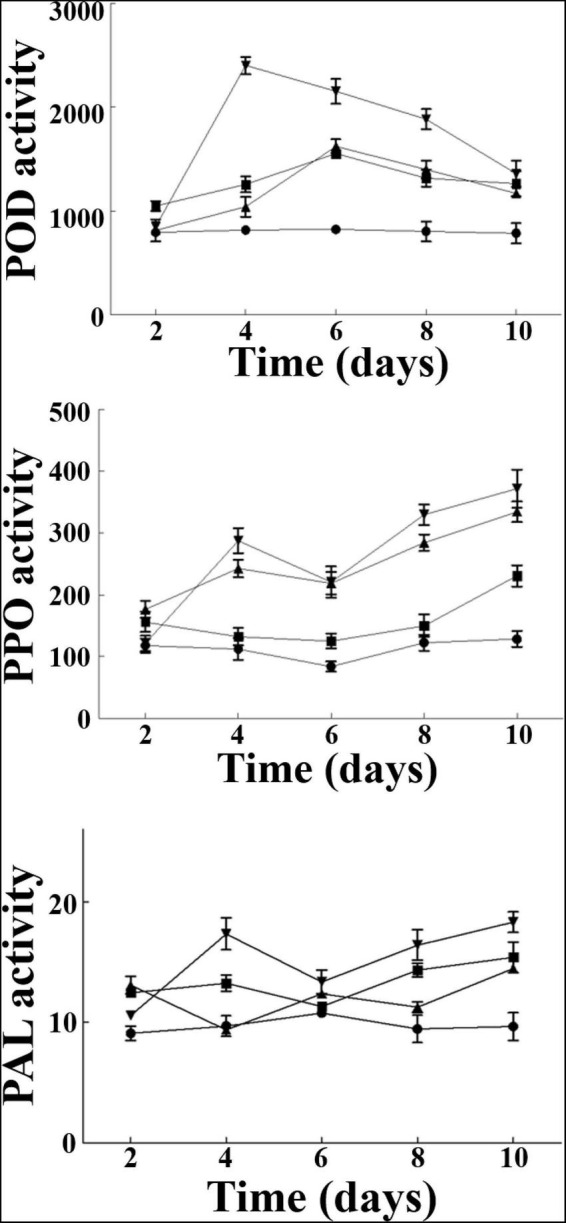
Effect of *B. siamensis* YC-9 on POD, PPO, and PAL activities (U/g⋅Fw⋅h) of cucumber root. A(●): CK; B(■): only YC-9 suspension were inoculated; C(▲): only FOC suspension were inoculated; D(▼): challenge inoculation with FOC suspension after 48 h of YC-9 suspension inoculation. Each data point represents the mean ± standard deviation of three replicates in each treatment group. Error bars represent the standard deviation from three replicates.

The resistance-related enzymes (POD, PPO, and PAL) activities in the roots of cucumber seedlings were measured at different times after pathogen inoculation (Figure 4). The results showed that the POD activity of cucumber treated with *B. siamensis* YC-9 was significantly higher than other treatments after pathogen inoculation and increased to the top level of 2,401 U/g⋅Fw⋅h on the 4th day, while the group A (CK), group B (only *B. siamensis* YC-9 was inoculated), and group C (only FOC was inoculated) were only 818, 1,244, and 1,024 U/g⋅Fw⋅h, respectively. The activities of PPO and PAL after pathogen inoculation were the overall upward trend, and their activities were maximum on the 10th day with 380 and 18.6 U/g⋅Fw⋅h, respectively. The results showed that *B. siamensis* YC-9 treatment could significantly increase the activities of PAL, PPO, and POD for some period of time after treatment in contrast to other treatments.

### Amplification of Antibiotics Encoding Genes

The presence of genes for biosynthesis of antibiotics such as 2,4-diacetyl phloroglucinol (DAPG), pyrrolnitrin (PRN), bacillomycin, fengycin, iturin, and surfactin in *B. siamensis* YC-9 was confirmed by PCR analysis with gene-specific primers. The primer groups and PCR amplification conditions were listed in Table 1. The primer set SRFAF/R amplified a 1,300-bp fragment from the *srf*AA gene, involved in the biosynthesis of surfactin. The primer set FEND1F/R amplified a 964-bp fragment from the *fen*D gene, involved in the biosynthesis of fengycin. The primer set BACC1F/R amplified an 875-bp fragment from the *bmy*B gene, involved in the biosynthesis of bacillomycin. The primer set ItuC-F/R amplified a 465-bp fragment from *itu*C gene, involved in the biosynthesis of iturin. The primer sets for Phl2a and Phl2b amplified a 745-bp fragment from *phl*D gene, involved in the biosynthesis of DAPG. The primer sets for PrnCf/r amplified a 719-bp fragment from the *prn*C gene, involved in the biosynthesis of PRN. The results of electrophoresis showed that *B. siamensis* YC-9 contained genes for biosynthesis of bacillomycin, iturin, and surfactin (Supplementary Figure 5).

### Separation and Identification of the Secondary Metabolites From *Bacillus siamensis* YC-9

**FIGURE 5:**
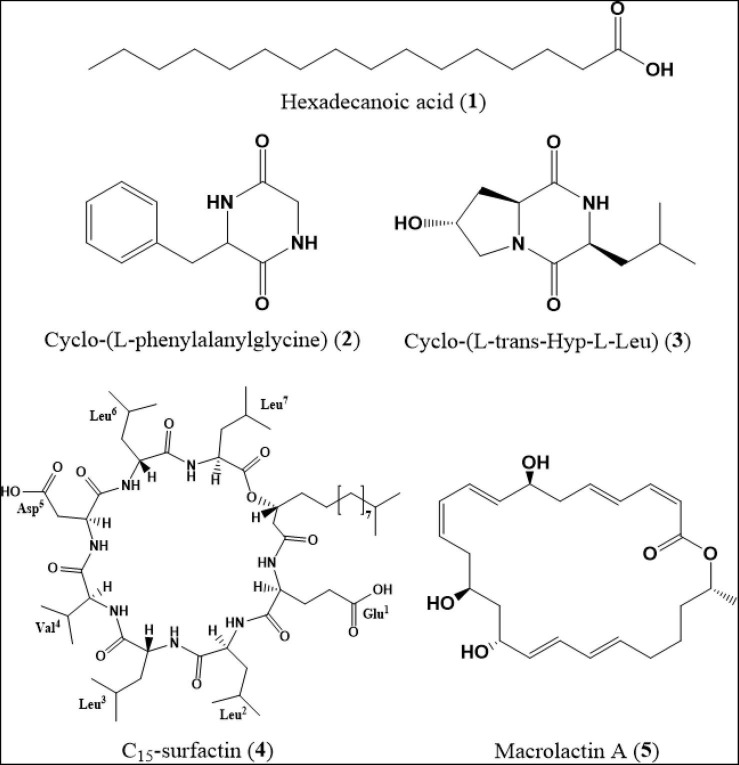
Chemical structures of secondary metabolites (**1–5**).

Chromatographic separation of the ethyl acetate extracts from the filtrate of *B. siamensis* YC-9 afforded five secondary metabolites (Figure 5), which were identified as hexadecanoic acid (**1**) (Aiyelaagbe et al., 2019), cyclo-(L-phenylalanylglycine) (**2**) (Zhuravleva et al., 2011), cyclo-(L-trans-Hyp-L-Leu) (**3**) (Xiang et al., 2020), C_15_-surfactin (**4**) (Tang et al., 2007), and macrolactin A (**5**) (Schneider et al., 2007) by spectroscopic data (Supplementary Figures 6–10) analyses, and the comparison of their derivative data in the literature.

### Antifungal Activity of Secondary Metabolites From *Bacillus siamensis* YC-9

**FIGURE 6:**
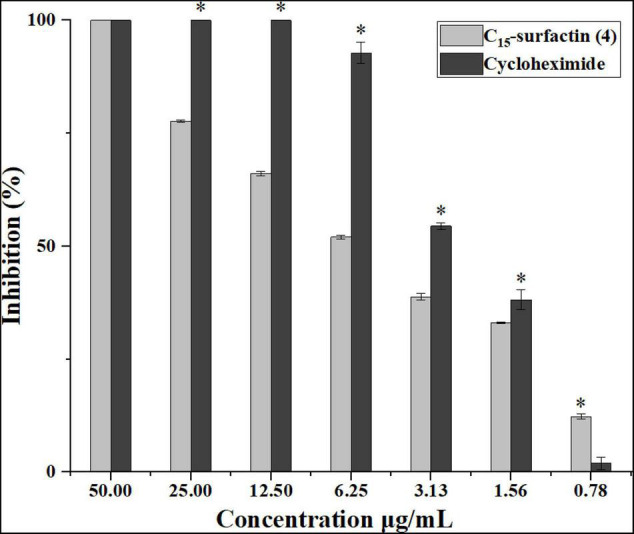
Effect of compound (**4**) (C_15_-surfactin) and referenced cycloheximide on conidia germination of FOC. Asterisks indicate a significant difference between the control (cycloheximide) and compound (**4**) treated conidia (*t*-test; *P < 0.05). Error bars represent the standard deviation from three replicates.

The isolated secondary metabolites from the culture of strain YC-9 were further evaluated for their antifungal activities by using the spore germination inhibition assay. Figure 6 showed C_15_-surfactin (**4**) had significantly antifungal activity against spore germination of FOC when compared to cycloheximide co-assayed as a positive reference and had higher antifungal activity with the increasing concentration. The calculated IC_50_ value of antifungal activity for C_15_-surfactin (**4**) was 5.1 μg/ml, which was comparable to that of the positive control, cycloheximide (IC_50_ value of 2.6 μg/ml). However, the other secondary metabolites were not active against spore germination (IC_50_ > 50 μg/ml).

## Discussion

Insect-associated microbiota had enormous potential to discover novel therapeutic agents against pathogens (Jang and Kikuchi, 2020). In fact, a recent comprehensive survey among diverse insects of 15 orders revealed that insect-associated *Streptomyces* inhibited more antimicrobial-resistant bacteria than soil-derived *Streptomyces* (Chevrette et al., 2019). As a biological material, termite nest is an important microbial resource. Due to its unique environment, the bacteria in a termite nest are more likely to have good antimicrobial activity than those in a normal environment (Long et al., 2010). For example, *Neonectria discophora* SNB-CN63 isolated from a termite nest has strong antimicrobial activity (Nirma et al., 2015). We isolated and screened a termite nest associated with *B. siamensis* YC-9 with significant antagonistic activity against FOC, which indicated the possibility that neglected insect symbiotic microorganisms could be a good source for microbial biocontrol agents.

Many genera of bacteria have latent capacity as biological control agents (BCAs), including genera of *Bacillus*, *Azospirillum*, *Pseudomonas*, *Burkholderia*, and *Enterobacter* (Xie et al., 2021). Species of *Bacillus* are the most commonly commercialized biocontrol agents due to their high tolerance of environmental stress, plant-growth promoting properties, and production of antimicrobial compounds (Khan et al., 2020; Rojas-Solis et al., 2020). Among the 30 bacterial isolates in this study, *B. siamensis* YC-9 displayed potent antifungal activity against FOC in *in vitro* experiments, exhibiting an inhibition rate of 72.5%. The results of the further pot test demonstrated that an application of *B. siamensis* YC-9 prior to inoculation of roots with FOC could significantly reduce both the incidence and severity of cucumber *Fusarium* wilt. Although the results of the pot experiment cannot predict the outcome of field trials, the demonstrated ability to reduce disease severity indicates that *B. siamensis* YC-9 has a promising potential for use under field conditions.

Plant growth-promoting bacteria (PGPB) can enhance plant yield and control phytopathogens, constituting the most widely studied and increasingly used tool in modern agriculture (Emmanuel and Babalola, 2020). *Bacillus* is one of the more widely studied PGPB, which is partly due to its production of ACC deaminase, indole-3-acetic acid (IAA), organic acids, siderophores, and phosphate solubilization (Khan et al., 2020; Shin et al., 2021). Our study showed that the application of *B. siamensis* YC-9 significantly increased the dry weight, fresh weight, plant height, and root length of cucumbers in pot experiments. Therefore, insect-associated microbes may be an important source of PGPB. However, the mechanism of promoting growth needs to be further examined.

Microbial-induced systemic resistance is an important aspect of the biological control of plant diseases. Enzymes such as PPO, PAL, and POD are associated with systemic-induced resistance in plant tissues (Qiu et al., 2022). In this study, *B. siamensis* YC-9 was demonstrated to have the ability to enhance the activity of POD, PAL, and PPO in cucumber roots when the seedlings were challenged with FOC, which contributed to a reduction in disease severity. Although many studies have shown that *B. siamensis* could promote plant growth, induce host resistance, and improve plant tolerance to abiotic stress (Lee et al., 2018; Xie et al., 2021), few studies have been conducted on the use of *B. siamensis* to prevent and treat cucumber diseases, especially cucumber *Fusarium* wilt. To the best of our knowledge, this is the first report on the use of *B. siamensis* to induce systemic resistance against FOC in the cucumber seedling roots.

The production of antifungal metabolites may be the most famous and important mechanism used by biocontrol bacteria to limit the pathogens’ invasion into host plant tissues. For example, lipopeptide production played a major role in the successful control of cucumber *Fusarium* wilt by *B. subtilis* SQR 9 (Cao et al., 2012). Bacillomycin is another member of the iturin family produced by bacilli with a strong antifungal spectrum (Caulier et al., 2019). Purified surfactins can also inhibit the growth of some fungi (Ma et al., 2020; Li et al., 2021). A PCR was used to determine the antibiosis mechanisms of *B. siamensis* YC-9 by screening six genes involved in the biosynthesis of antibiotics. Amplicons of the expected sizes were detected as *itu*C for iturin, *srf*AA for surfactin, and *bmy*B for bacillomycin synthesis. These findings suggest that *B. siamensis* YC-9 could produce multiple antibiotics as a biocontrol agent. Due to the limitations of separation methods, we only isolated C_15_-surfactin and related metabolites in the stage of separating secondary metabolites.

## Conclusion

The use of *B. siamensis* YC-9 isolated from the termite nest was shown to have potential for the prevention and control of cucumber *Fusarium* wilt, which indicated the possibility that neglected insect symbiotic microorganisms could be a good source for microbial biocontrol agents. The biological control ability of *B. siamensis* YC-9 against FOC appeared to be due to several mechanisms, including the induction of host resistance and the secretion of antifungal metabolites. The bioactive compound C_15_-surfactin was successfully identified from the ethyl acetate extract of fermentation filtrate. It could significantly inhibit the germination of FOC spores, and the effect was comparable to that of the positive control, cycloheximide. A pot experiment also proved that *B. siamensis* YC-9 had an obvious growth-promoting effect on cucumber seedlings. Therefore, the termite-nest-associated *B. siamensis* YC-9 could be a potential biological control agent for integrated control of soil-borne diseases such as cucumber *Fusarium* wilt.

## Data Availability Statement

The original contributions presented in the study are included in the article/Supplementary Material, further inquiries can be directed to the corresponding author.

## Author Contributions

YZ and KK designed the research. YZ supervised the study. LZ performed the experiments, analyzed the data, and wrote the manuscript. JW performed the experiments and analyzed the results. All authors revised the manuscript and approved the final version for submission.

## Conflict of Interest

The authors declare that the research was conducted in the absence of any commercial or financial relationships that could be construed as a potential conflict of interest.

## Publisher’s Note

All claims expressed in this article are solely those of the authors and do not necessarily represent those of their affiliated organizations, or those of the publisher, the editors and the reviewers. Any product that may be evaluated in this article, or claim that may be made by its manufacturer, is not guaranteed or endorsed by the publisher.
