# Supplementary material for: Termite Nest Associated Bacillus siamensis YC-9 Mediated Biocontrol of Fusarium oxysporum f. sp. cucumerinum
Source: Front Microbiol. 2022 Jun 1;13:893393. doi: 10.3389/fmicb.2022.893393 (PMC9198579; doi:10.3389/fmicb.2022.893393)
Supplement: Supplementary file 1 [file Presentation_1.pdf]

Antifungal activity of the termite nest associated *Bacillus siamensis* YC-9 against *Fusarium oxysporum* f. sp. *cucumerinum* and the identification of antifungal metabolite

Lingfeng Zhou[1,†], Junyong Wang[1,†], Fei Wu[1], Caiping Yin[1], Ki Hyun Kim[2], Yinglao Zhang[1,*]

[1]*College of Life Sciences, Anhui Agricultural University, Hefei 230036, People's Republic of China*

[2]*School of Pharmacy, Sungkyunkwan University, Suwon 16419, Republic of Korea*

*Correspondence:

E-mail address: zhangyl@ahau.edu.cn, Tel.: +86-551-657862169

†These authors have contributed equally to this work.

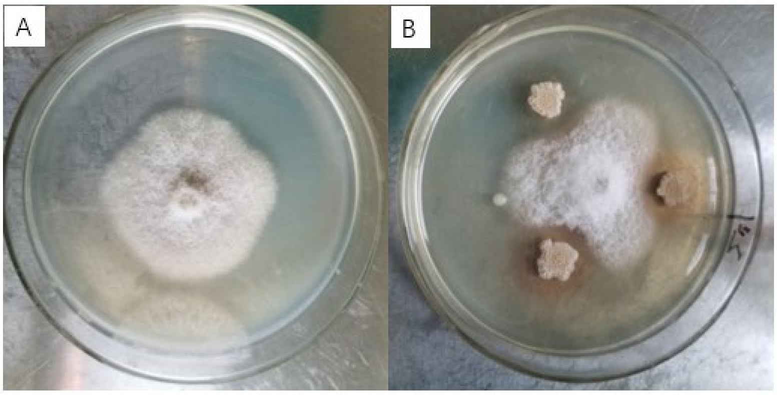

Fig. S1 Antagonistic interaction between YC-9 and FOC. A: control (only FOC). B: Inhibition effect of strain YC-9 against FOC.

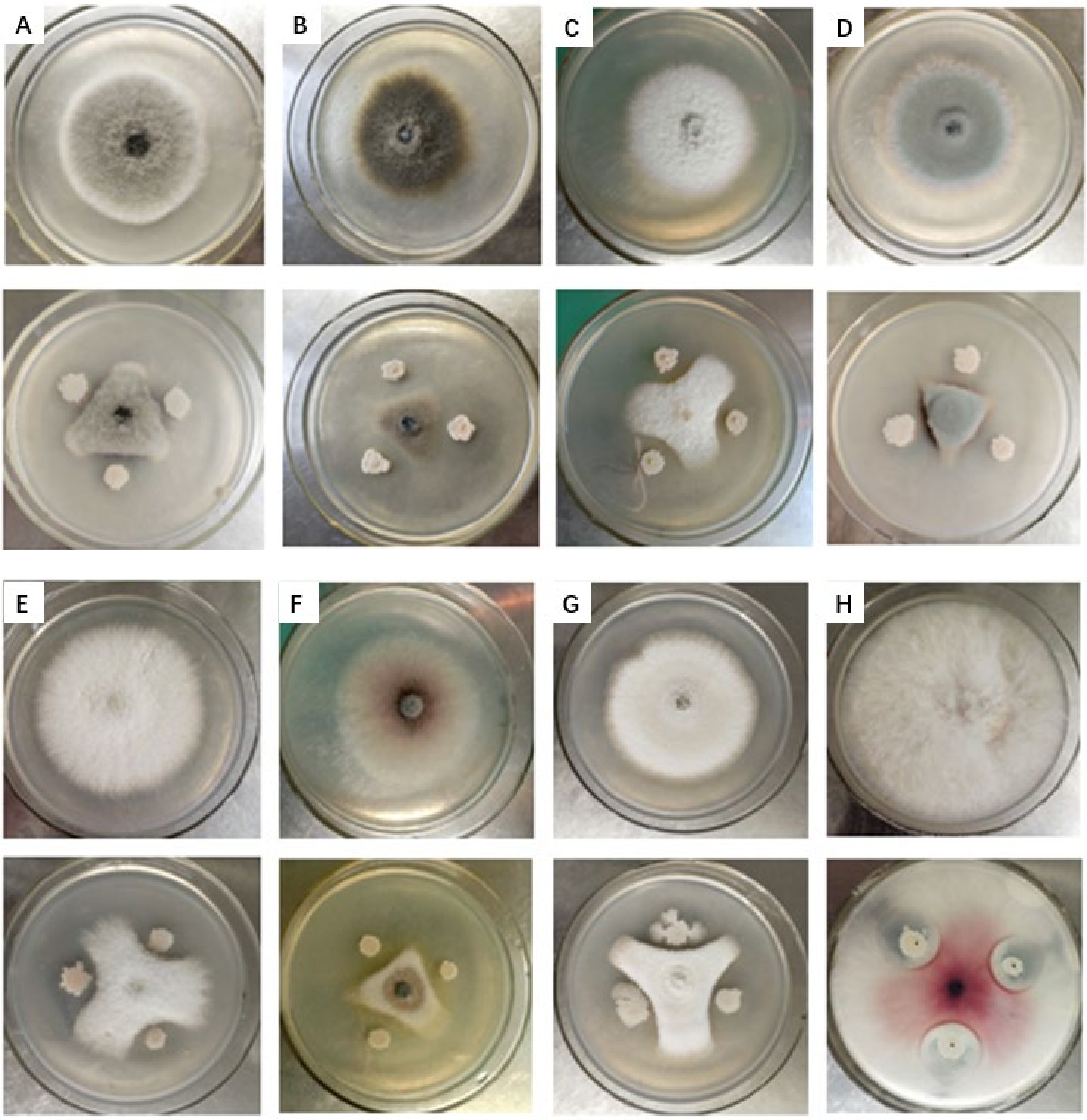

Fig.S2 Inhibition effect of strain YC-9 against plant pathogenic fungi. A: *C. cassiicola*; B: *B. cinerea*; C: *F. oxysporum* f. sp.*vasinfectum*; D: *C. graminicola*; E: *F. oxysporum* f. sp. *Mornordicae*; F: *A. solani*; G: *C. lunata*; H: *F. graminearum*.

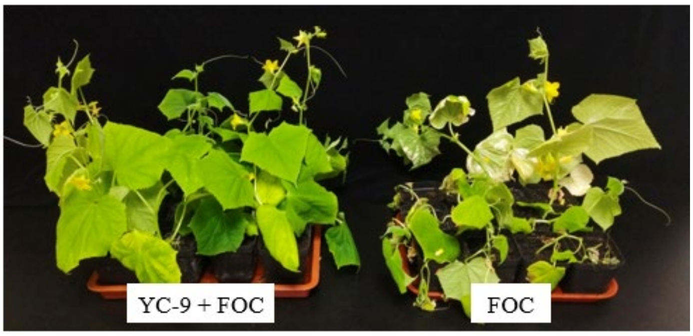

Fig. S3 Potted activity of YC-9 against FOC

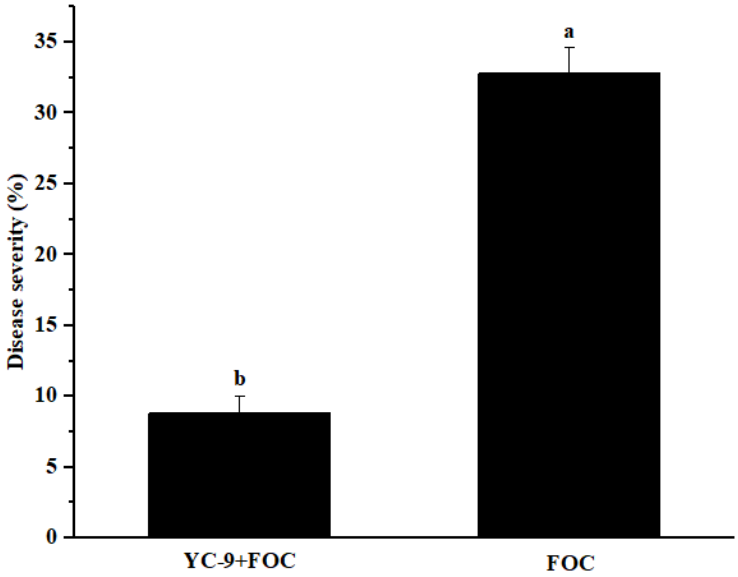

Fig. S4 Reduction of disease severity upon application of strain YC-9 during *in vivo* challenge experiment in the presence of conidial suspension of FOC.

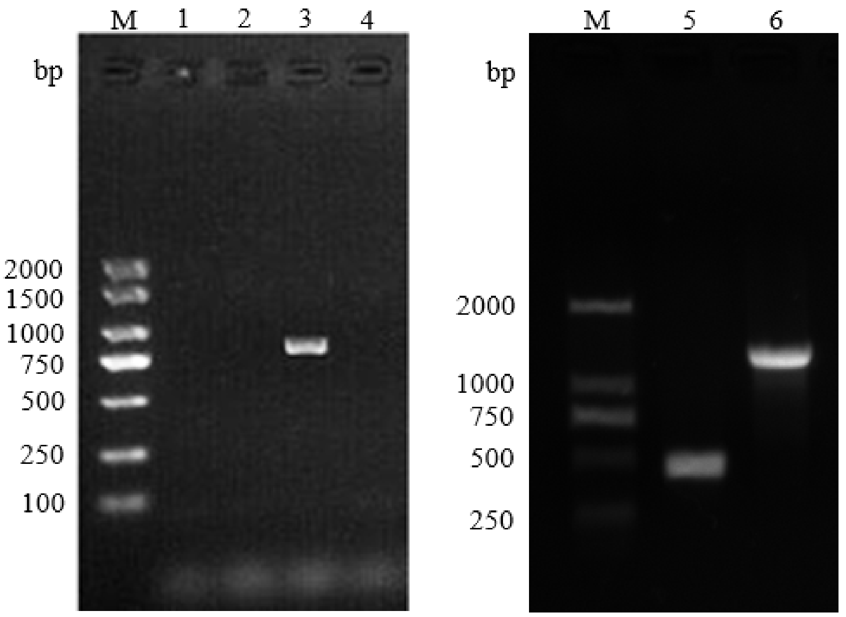

Fig. S5 PCR of antibiotic synthesis genes of YC-9 strain. M: Marker. 1: 2, 4-diacetyl phloroglucinol. 2: Pyrrolnitrin. 3: Bacillomycin. 4: Fengycin. 5: Iturin. 6: Surfactin.

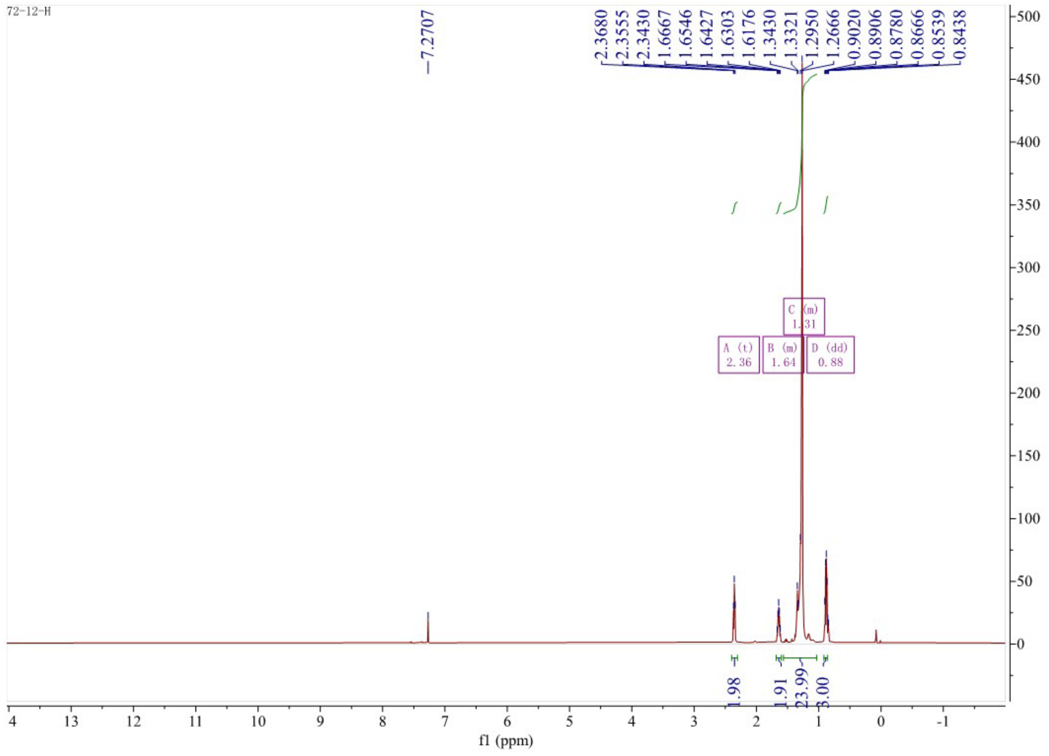

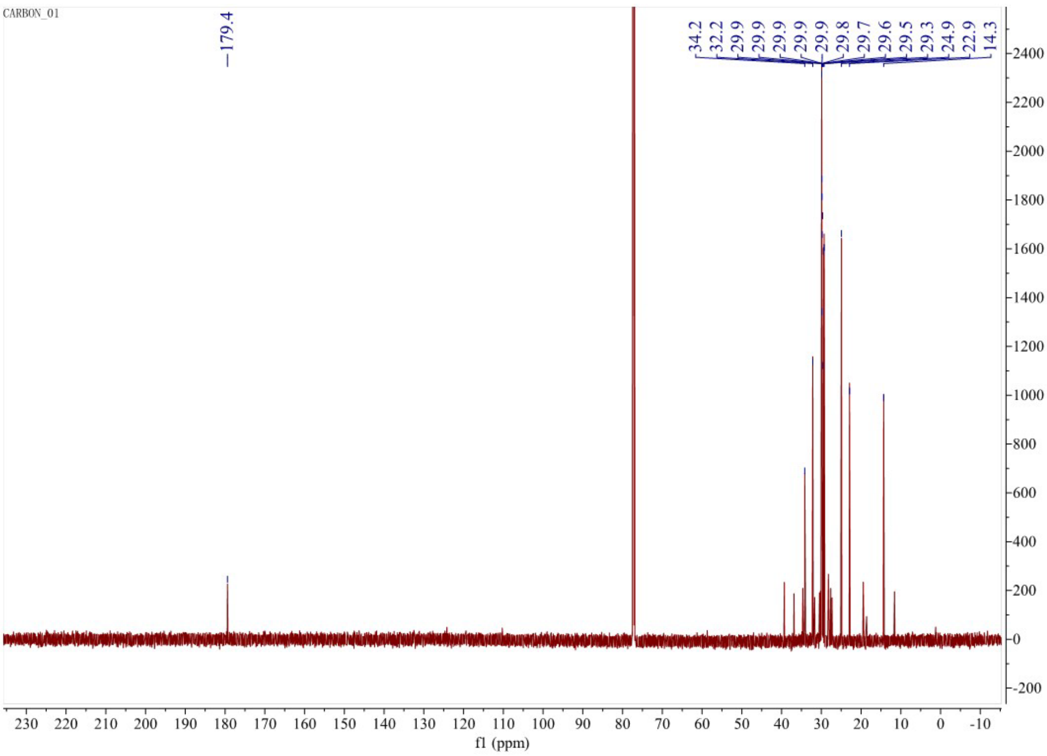

Sample Name	Sample17	Position	P1-B6	Instrument Name	Instrument 1
User Name		Inj Vol	5	InjPosition	
Sample Type	Sample	IRM Calibration Status	Success	Data Filename	ZYL-20210922--0017.d
ACQ Method	200-1500-FU.m	Comment		Acquired Time	9/22/2021 11:53:41 AM (UTC+08:00)

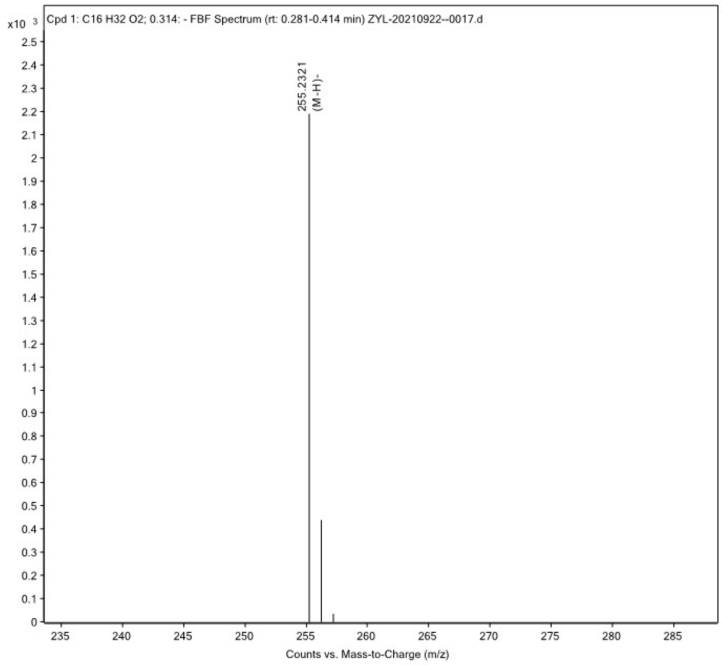

Fig. S6 ^{1}H NMR, ^{13}C NMR and mass spectral data of compound **1**

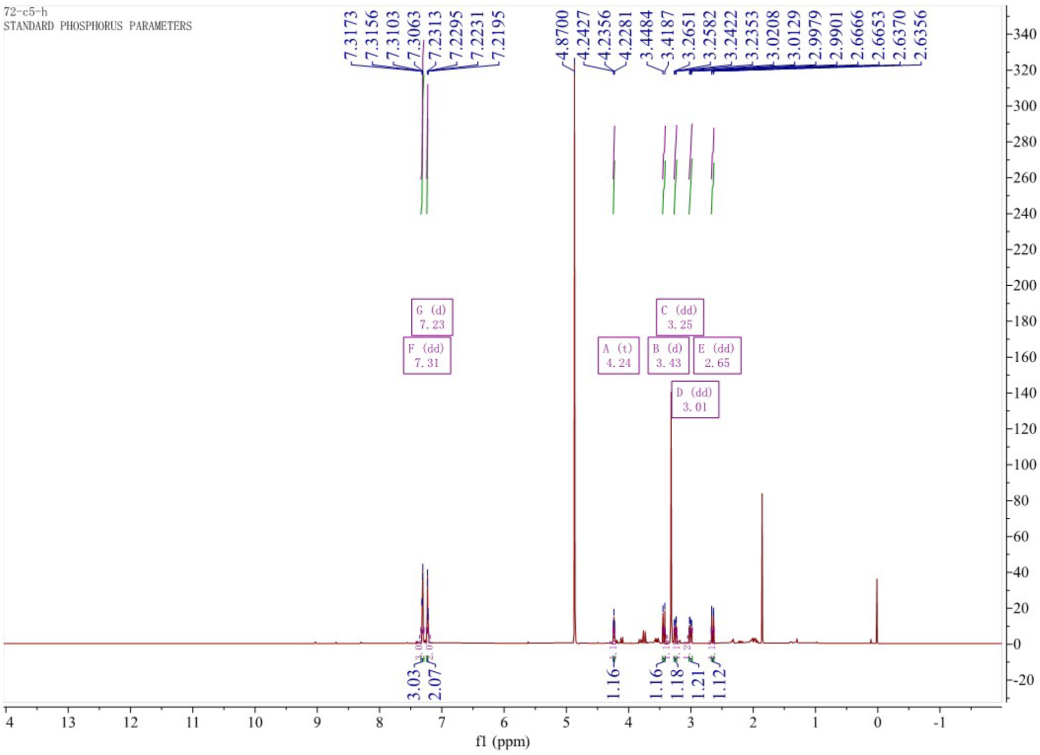
72-c5-h
STANDARD PHOSPHORUS PARAMETERS
7.3173
7.3156
7.3103
7.3063
7.2313
7.2295
7.2231
7.2195
4.8700
4.2427
4.2356
4.2281
3.4484
3.4187
3.2651
3.2582
3.2422
3.2353
3.0208
3.0129
2.9979
2.9901
2.6666
2.6653
2.6370
2.6356
G (d) 7.23
F (dd) 7.31
A (t) 4.24
B (d) 3.43
C (dd) 3.25
D (dd) 3.01
E (dd) 2.65
3.03
2.07
1.16
1.16
1.18
1.21
1.12
f1 (ppm)

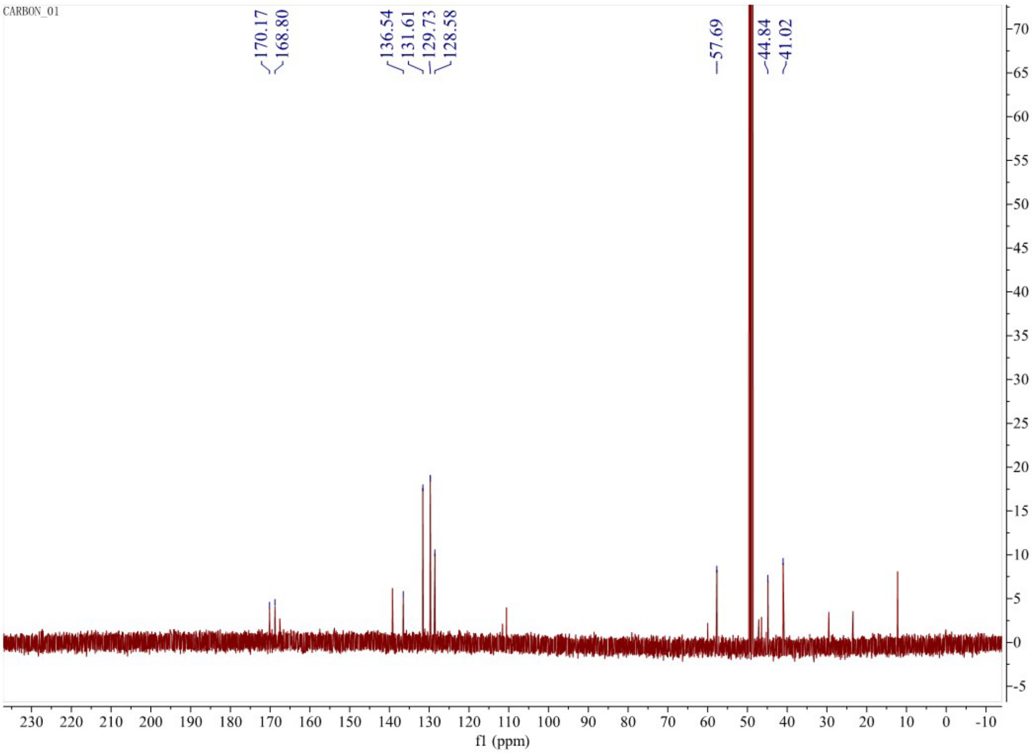
CARBON_01
170.17
168.80
136.54
131.61
129.73
128.58
57.69
44.84
41.02
f1 (ppm)

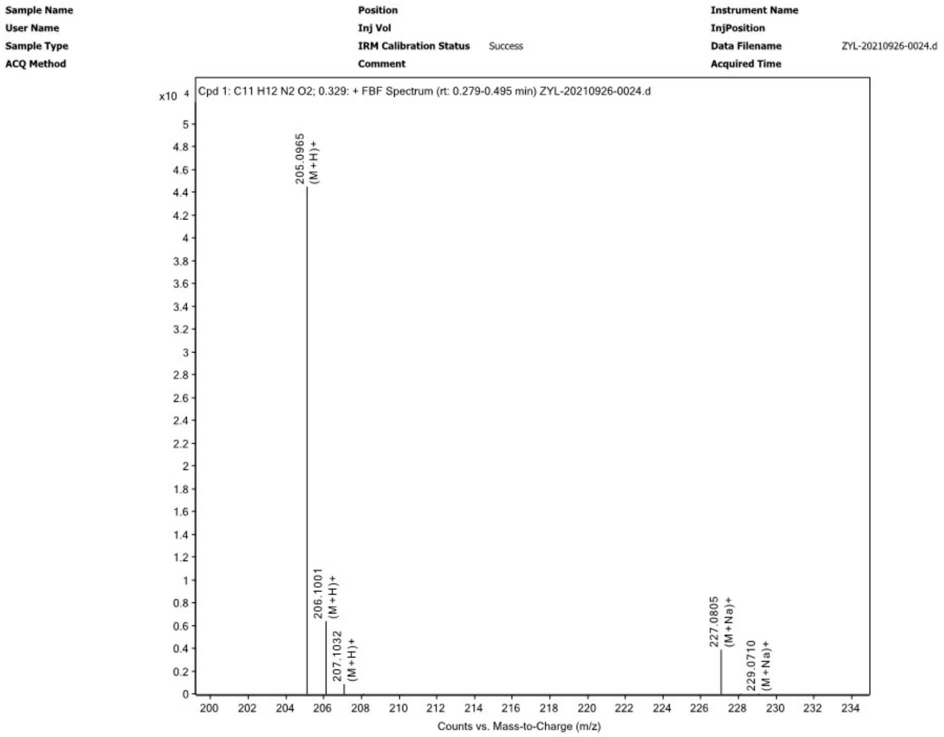

Fig. S7 ^{1}H NMR, ^{13}C NMR and mass spectral data of compound **2**

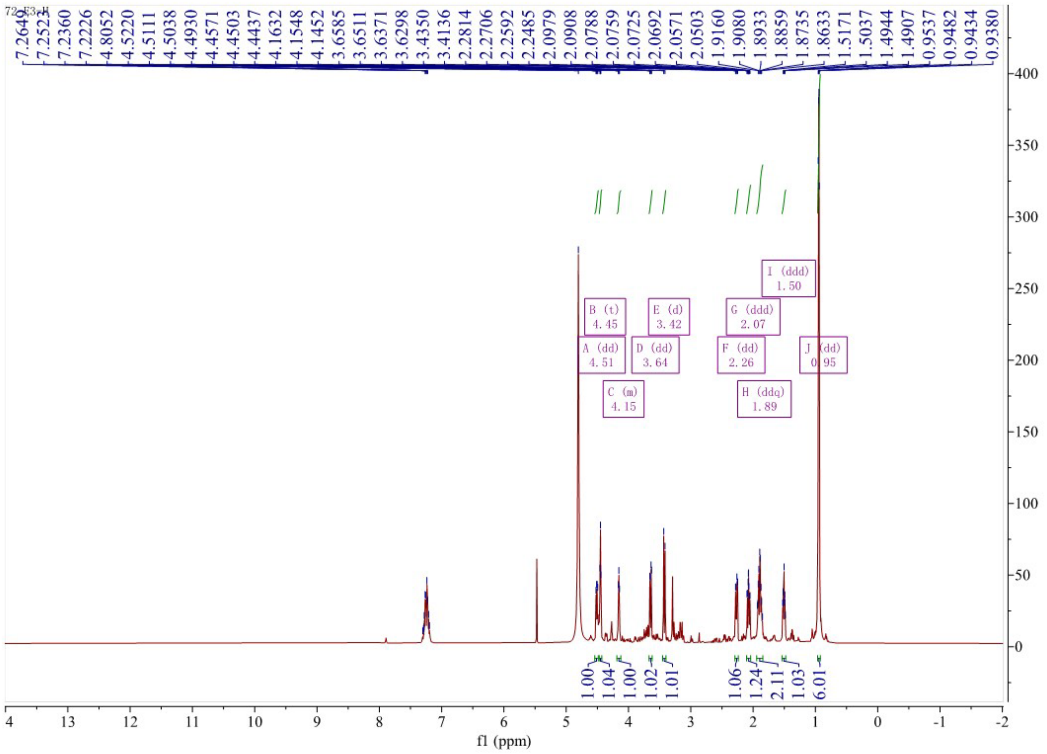

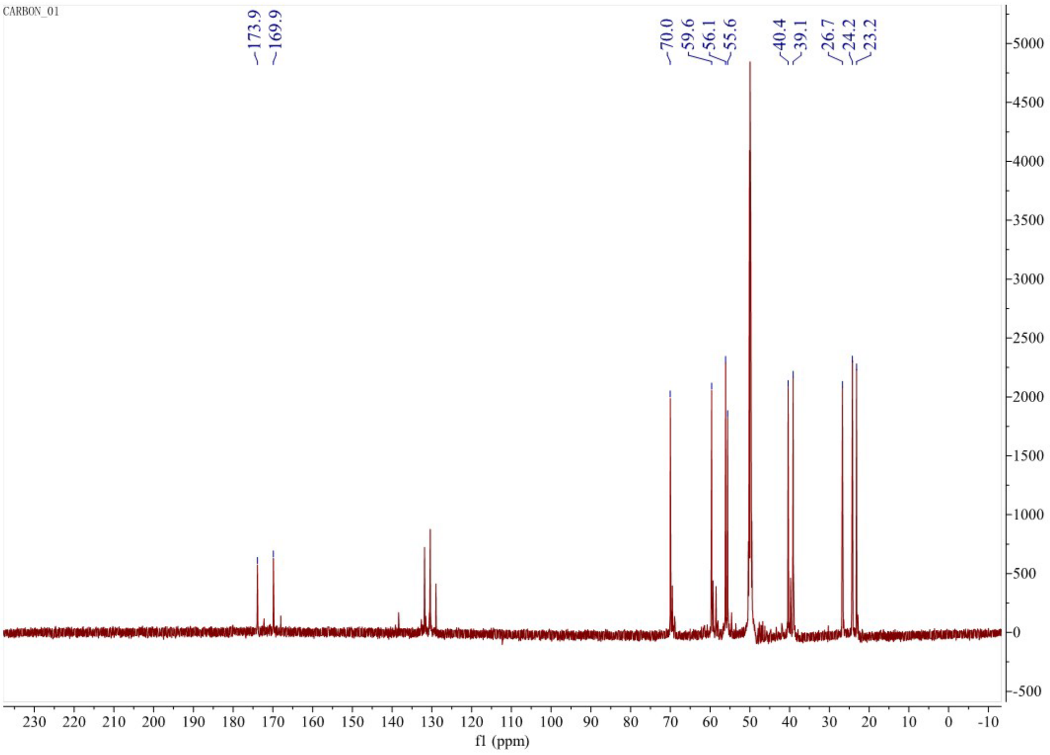

**Sample Name**	Sample26	**Position**	P2-C4	**Instrument Name**	Instrument 1
**User Name**		**Inj Vol**	1	**InjPosition**	
**Sample Type**	Sample	**IRM Calibration Status**	Success	**Data Filename**	ZYL-20210926-0026.d
**ACQ Method**	150-1500-CH3CN.m	**Comment**		**Acquired Time**	9/26/2021 5:20:34 PM (UTC+08:00)

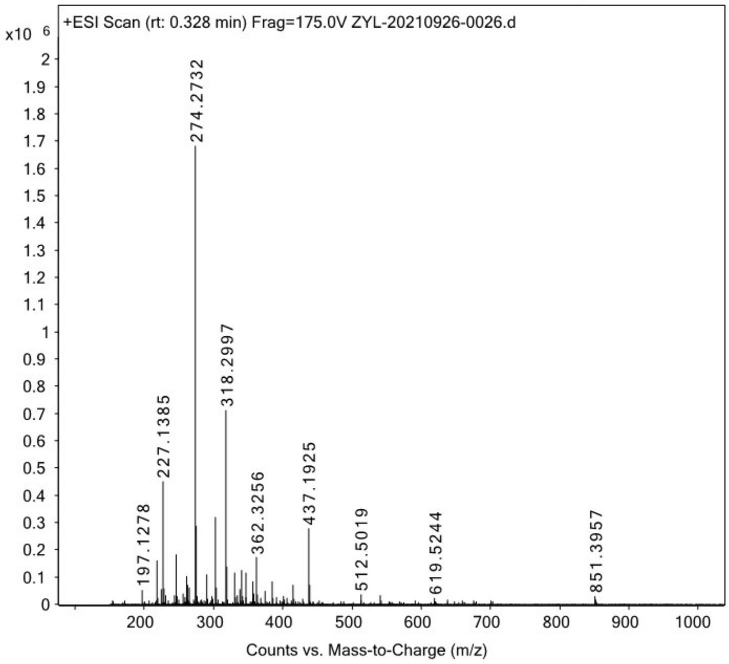

Fig. S8 ^{1}H NMR, ^{13}C NMR and mass spectral data of compound **3**

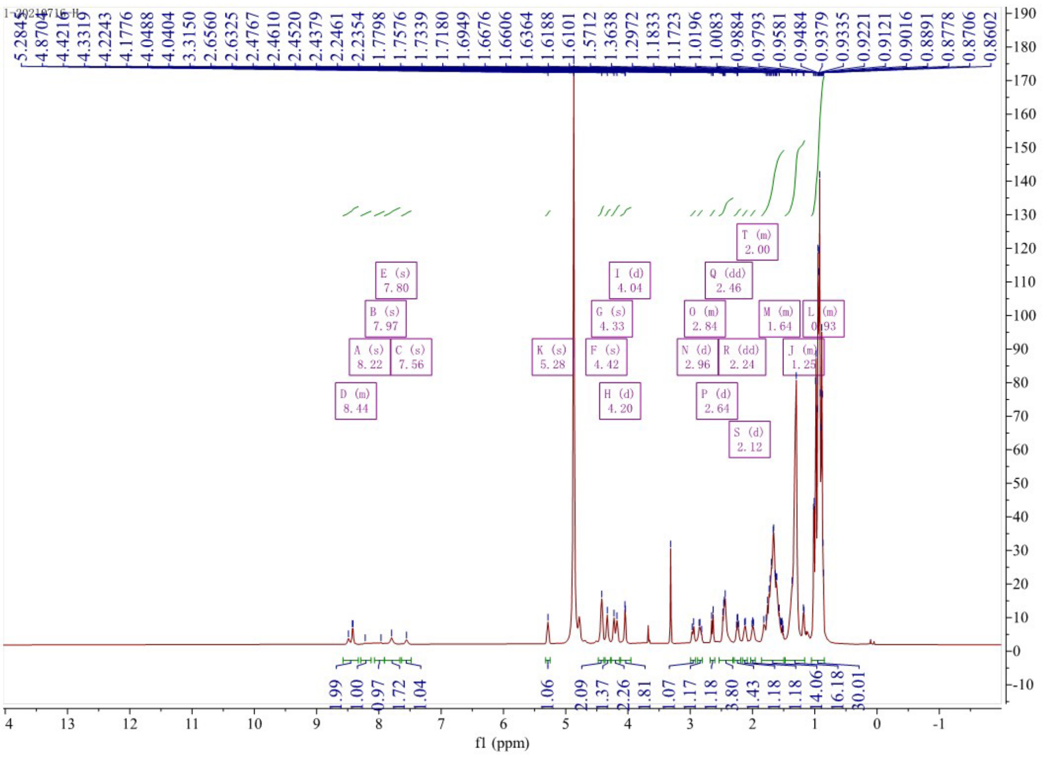
5.2844 4.8704 4.4216 4.3319 4.2243 4.1776 4.0488 4.0404 3.3150 2.6560 2.6325 2.4767 2.4610 2.4520 2.4379 2.2461 2.2354 1.7798 1.7576 1.7339 1.7180 1.6949 1.6676 1.6606 1.6364 1.6188 1.6101 1.5712 1.3638 1.2972 1.1833 1.1723 1.0196 1.0083 0.9884 0.9793 0.9581 0.9484 0.9379 0.9335 0.9221 0.9121 0.9016 0.8891 0.8778 0.8706 0.8602
T (m) 2.00
E (s) 7.80
I (d) 4.04
Q (dd) 2.46
B (s) 7.97
G (s) 4.33
O (m) 2.84
M (m) 1.64
L (m) 0.93
A (s) 8.22
C (s) 7.56
K (s) 5.28
F (s) 4.42
N (d) 2.96
R (dd) 2.24
J (m) 1.25
D (m) 8.44
H (d) 4.20
P (d) 2.64
S (d) 2.12
1.99 1.00 0.97 1.72 1.04 1.06 2.09 1.37 2.26 1.81 1.07 1.17 1.18 3.80 1.43 1.18 1.18 14.06 16.18 30.01
f1 (ppm)

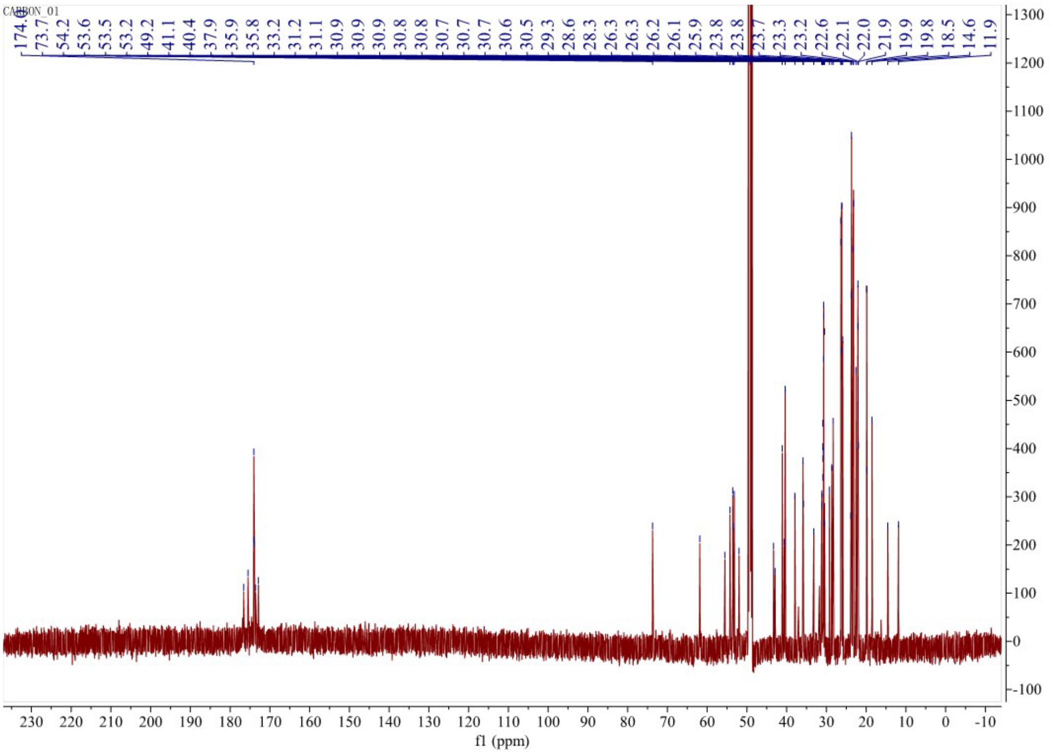
CARBON_01
174.0 73.7 54.2 53.6 53.5 53.2 49.2 41.1 40.4 37.9 35.9 35.8 33.2 31.2 31.1 30.9 30.9 30.9 30.8 30.7 30.7 30.7 30.6 30.5 29.3 28.6 28.3 26.3 26.3 26.2 26.1 25.9 23.8 23.8 23.7 23.3 23.2 22.6 22.1 22.0 21.9 19.9 19.8 18.5 14.6 11.9
f1 (ppm)

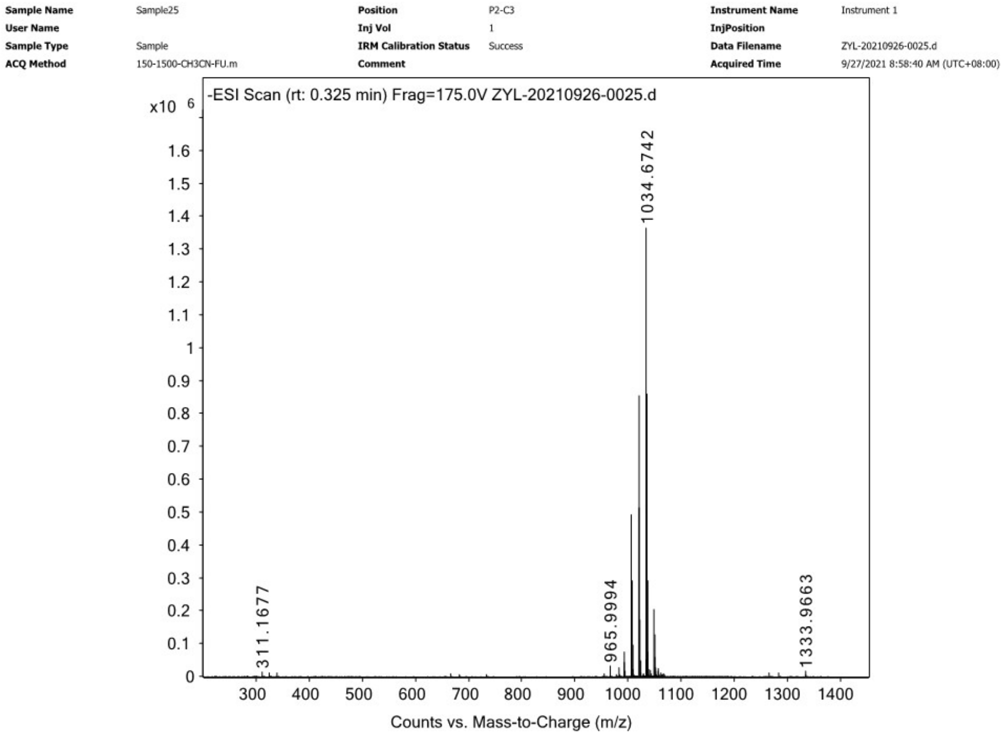

Fig. S9 ^{1}H NMR, ^{13}C NMR and mass spectral data of compound **4**

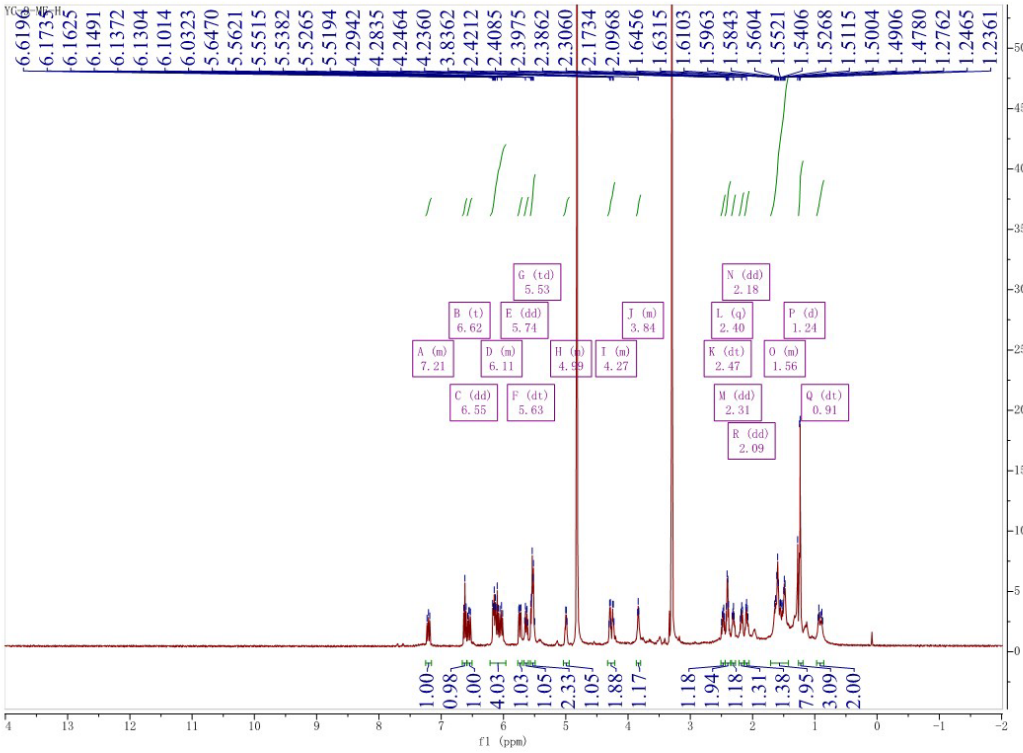

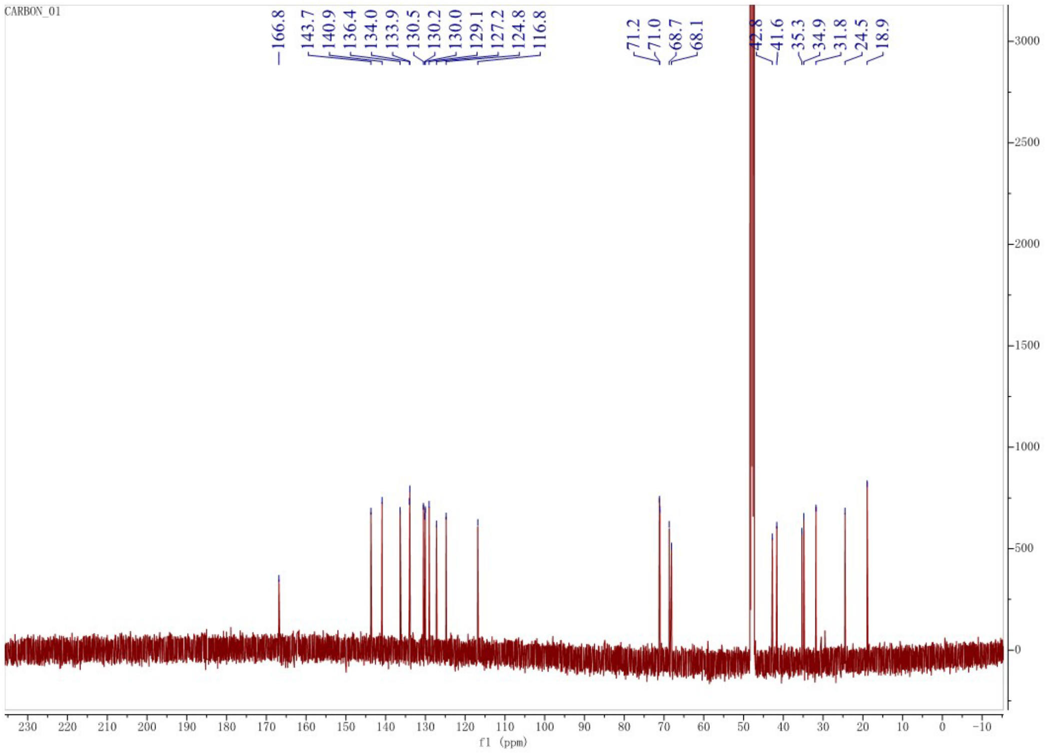

**Sample Name**	Sample1	**Position**	P2-D2	**Instrument Name**	Instrument 1
**User Name**		**Inj Vol**	1	**InjPosition**	
**Sample Type**	Sample	**IRM Calibration Status**	Success	**Data Filename**	ZYL-20220110-0001.d
**ACQ Method**	ESI-90%-MeOH-(+)-(100-1000)-1min.m	**Comment**		**Acquired Time**	1/10/2022 11:17:43 AM (UTC+08:00)

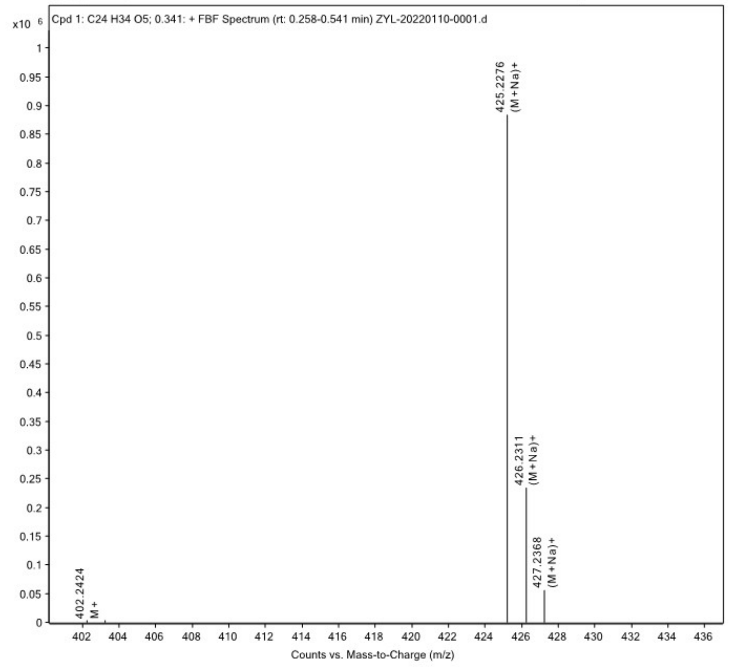

Fig. S10 ^{1}H NMR, ^{13}C NMR and mass spectral data of compound **5**
